# Health Behaviors and Lifestyle Interventions in African American Breast Cancer Survivors: A Review

**DOI:** 10.3389/fonc.2019.00003

**Published:** 2019-01-22

**Authors:** Raheem J. Paxton, William Garner, Lorraine T. Dean, Georgiana Logan, Kristen Allen-Watts

**Affiliations:** ^1^Department of Community Medicine and Population Health, The University of Alabama, Tuscaloosa, AL, United States; ^2^Department of Life and Health Sciences, University of North Texas at Dallas, Dallas, TX, United States; ^3^Department of Epidemiology, John Hopkins School of Public Health, Baltimore, MD, United States

**Keywords:** African American, breast cancer, cancer survivor, cancer survivorship, diet, physical activity, review, obesity

## Abstract

**Background:** African American breast cancer survivors have a higher incidence of estrogen receptor negative and basal-like (e.g., triple negative) tumors, placing them at greater risk for poorer survival when compared to women of other racial and ethnic groups. While access to equitable care, late disease stage at diagnosis, tumor biology, and sociodemographic characteristics contribute to health disparities, poor lifestyle characteristics (i.e., inactivity, obesity, and poor diet) contribute equally to these disparities. Lifestyle interventions hold promise in shielding African American survivors from second cancers, comorbidities, and premature mortality, but they are often underrepresented in studies promoting positive behaviors. This review examined the available literature to document health behaviors and lifestyle intervention (i.e., obesity, physical activity, and sedentary behavior) studies in African American breast cancer survivors.

**Methods:** We used PubMed, Academic Search Premier, and Scopus to identify cross-sectional and intervention studies examining the lifestyle behaviors of African American breast cancer survivors. Identified intervention studies were assessed for risk of bias. Other articles were identified and described to provide context for the review.

**Results:** Our systematic review identified 226 relevant articles. The cross-sectional articles indicated poor adherence to physical activity and dietary intake and high rates of overweight and obesity. The 16 identified intervention studies indicated reasonable to modest study adherence rates (>70%), significant reductions in weight (range −1.9 to −3.6%), sedentary behavior (−18%), and dietary fat intake (range −13 to −33%) and improvements in fruit and vegetable intake (range +25 to +55%) and physical activity (range +13 to +544%). The risk of bias for most studies were rated as high (44%) or moderate (44%).

**Conclusions:** The available literature suggests that African American breast cancer survivors adhere to interventions of various modalities and are capable of making modest to significant changes. Future studies should consider examining (a) mediators and moderators of lifestyle behaviors and interventions, (b) biological outcomes, and (c) determinants of enhanced survival in this population.

## Introduction

The number of African American (AA) cancer survivors continues to increase (1). Despite this rapid increase, AAs are especially vulnerable to poor health outcomes. In particular, AA breast cancer survivors are at risk for second cancers and comorbid conditions that threaten their ability to live independently (2). Developing health promotion and disease prevention interventions for this population will reduce their risk for adverse outcomes following diagnosis. However, before developing such interventions, there is an urgent need to (a) understand the mechanisms that contribute to poor health outcomes and (b) review relevant health behavior and intervention studies that have been conducted in this population.

### Rationale

There are key indicators that contribute to cancer-related disparities between AA and non-Hispanic white (NHW) breast cancer survivors. Racial differences in tumor characteristics may contribute to the survival disparities between AA and NHW women (3–6). For example, several studies indicated that AA women were more likely to develop highly aggressive (7), “basal-like” tumors of the triple-negative subtype, but less likely than NHW women of the same ages to develop less aggressive, more treatable “luminal” tumors (8–17). While these data partially support the genetic admixture hypothesis or differences in tumor biology, socio-political constructs such as racism and segregation may be equally important (18). Several authors have indicated that AA women born in southern states with long-lasting vestiges of slavery (i.e., Jim Crow laws) (19, 20), as well as those exposed to persistent weathering from poor social environments are more likely than NHW women to be diagnosed with aggressive tumors (18, 21). Furthermore, others speculate that comorbidities are significant culprits behind the mortality disparities that exist between AA and NHW breast cancer survivors (22). In a historical cohort from the Henry Ford Health System, Tammemagi et al. (22) found that comorbidities accounted for nearly 50% of the overall and competing-cause survival disparity between AA and NHW women. The combination of these factors contributes to the health disparities that exist between AA and NHW breast cancer survivors.

The literature presented above, while informative, neglects critical elements that may shield AA breast cancer survivors from poor outcomes. These data assumes that health and wellness occur in a vacuum and that preventive behaviors such as diet and exercise are not relevant. As such, the body of literature describing AA cancer survivors has focused on differences in cancer-specific and overall outcomes but has failed to study extensively the factors that promote health.

### Objectives

Recent comprehensive reviews have documented the importance of healthy lifestyle factors in improving the health and well-being of cancer survivors (23–26). Lifestyle interventions hold promise in shielding African American survivors from second cancers, comorbidities, and premature mortality, but they are underrepresented in these studies. Studies that summarize the state of AA breast cancer survivorship may help to document the successes and opportunities that move the field forward. Therefore, the objectives of this review were to summarize published intervention studies documenting the efficacy or effectiveness of diet, exercise, and weight loss interventions in AA breast cancer survivors.

### Research Aims

The aims of this review were to (a) summarized key cohort, cross-sectional, and randomized trials; (b) examine published literature to document the efficacy or effectiveness of health behavior (i.e., diet, physical activity, and weight loss/maintenance) intervention studies conducted in AA in breast cancer survivors; and (c) propose strategies for moving the field of AA breast cancer survivorship forward. The information gained from this study may shed light on the strengths, weaknesses, and opportunities to enhance the health and well-being of AA breast cancer survivors.

## Methods

### Study Design

All types of study designs were identified and evaluated. The designs included cross-sectional, cohort, quasi-experimental, and randomized designs.

### Participants and Interventions

The participants were breast cancer survivors aged 18 years and older. However, we also included studies that focused exclusively on AA breast cancer survivors. The interventions were designed to improve physical activity and dietary intake and decrease sedentary behavior (i.e., prolonged sitting), dietary fat, or weight.

### Systematic Review Protocol

Although this is a general review study, we applied an approach similar to a systematic review to examine the articles. Two authors confirmed the inclusion and exclusion criteria as well as conducted the independent reviews. There were no disagreements that required a third party to adjudicate.

### Data Sources and Search Strategy

We searched three databases (PubMed, Academic Search Premier, and Scopus) with the following search terms–cancer survivor^*^, African American, breast neoplasm, and lifestyle behaviors (physical activity OR diet, OR sedentary behavior OR prolonged sitting OR weight loss OR weight management). The exclusion criteria were non-English language studies; theses or dissertations (not published in peer-reviewed journals); qualitative studies, and studies that did not report primary data for AA breast cancer survivors. We also excluded AA studies that focused exclusively on psychosocial variables (i.e., self-efficacy and quality of life) or those that did not have a focus on lifestyle behaviors as the primary independent or dependent variable. Also, we incorporated a separate search for seminal articles in the field that documented the state of cancer survivorship research. Many of these studies represented large randomized controlled trials, large cohort studies, or secondary analyses of these studies. The select studies identified were included in our search totals and used to guide our review of the literature. The time frame was limited from 1999 to October 2018. Select cross-sectional studies were reported on due to our emphasis on intervention studies.

### Data Analysis

The identified articles were used to create a flow diagram. The inclusion and exclusion criteria described above was used to determine the articles that were reviewed. The intervention studies were rated based on unique characteristics that determined the risk of bias. We selected 3 criteria to determine bias in the identified intervention studies. The criteria were based on the Cochrane Handbook for Systematic Reviews of Intervention (27). The criteria used included the (a) sample size, (b) study design, and (c) validity of the outcome measure. Sample sizes of < 30, 30 to 49, and 50 or higher were characterized as low, moderate, and high quality, respectively. Randomized trials were characterized as high quality, while other designs were characterized as low quality. Objectively assessed anthropometric, dietary, and physical activity outcomes were characterized as high quality, whereas self-reported outcomes were characterized as low quality. Each rating was transformed to a numerical, whereby scores of 11, 22, and 33 characterized as low, moderate, and high quality, respectively. Scores were summed across categories. Total scores of <60, 60 to <99, and 99 were characterized as high, moderate, and low bias studies.

## Results

### Study Selection Characteristics and Flow Diagram

**Figure 1:**
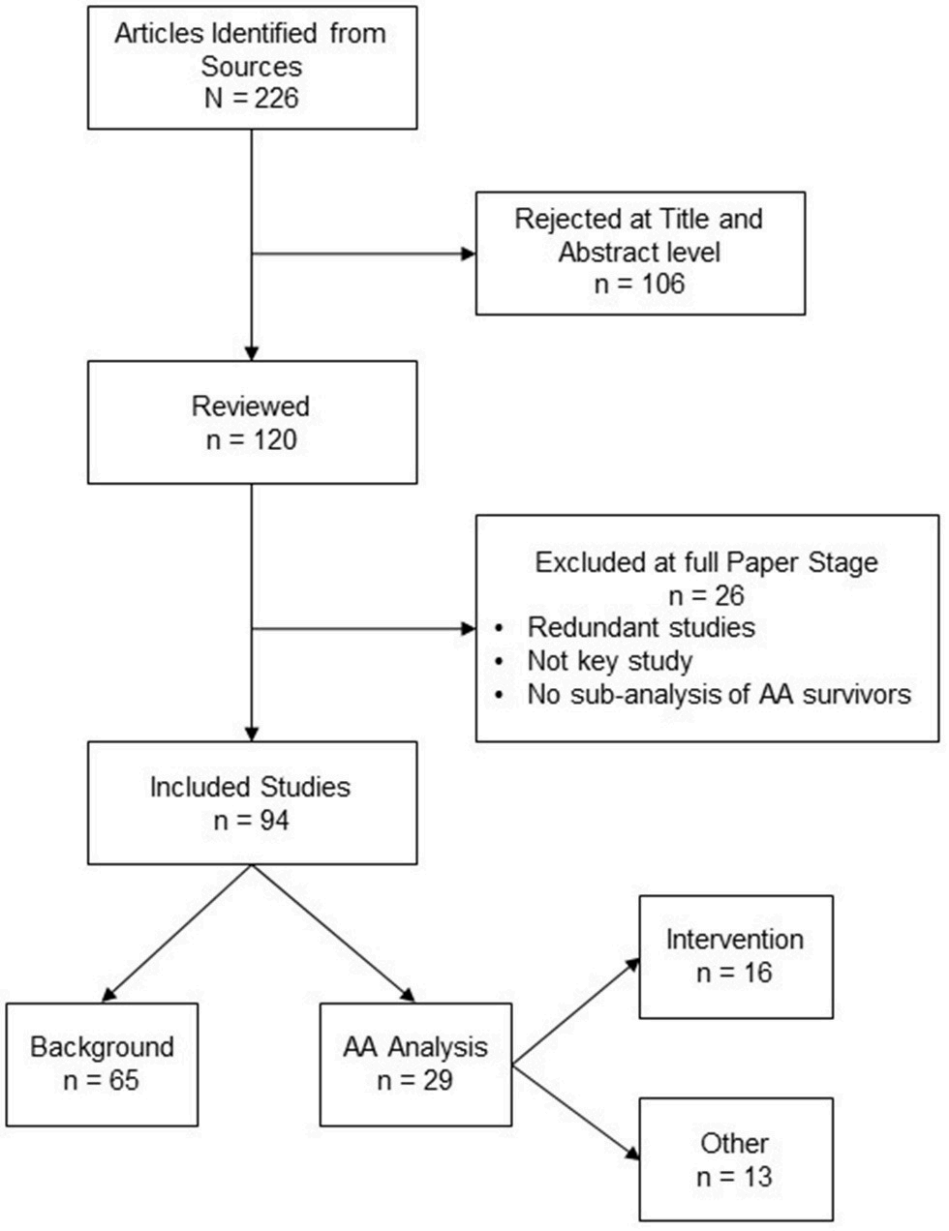
Diagram of the literature review and selection process.

A total of 226 total articles were identified. Some articles were redundant across databases or did not meet our inclusion criteria (*N* = 26). A total of 94 articles were reviewed comprehensively by our research team. Of the 94 articles identified, 65 pertained to seminal research in the field of cancer survivorship that provided a context for the 29 articles that were selected to characterize the health behaviors of AA breast cancer survivors. Sixteen of the 29 studies were unique intervention studies. Figure 1 depicts an image of our flow diagram.

### Synthesized Findings

#### Review of Lifestyle Factors in AA Breast Cancer Survivors

##### Obesity and weight gain

Studies have repeatedly shown that the relationship between body mass index (BMI) and breast cancer is not the same for AA and NHW women (28, 29). Excess weight gain after the age of 18 for AA women was associated with early onset (or premenopausal), estrogen-receptor negative, and triple negative breast cancers (30–33). Large cohort and national studies also indicate that AA breast cancer survivors have higher rates of obesity (body mass index [BMI] ≥30 kg/m^2^) (34–36), and gain more weight following treatment than women of other racial and ethnic groups (37).

Weight status and post-diagnosis weight gain may be the immediate causal factor in the poor prognostic outcomes experienced by AA women (38–40). Nichols et al. (41) found that obesity before diagnosis was associated with greater mortality in breast cancer survivors. Also, post-diagnosis weight gain of >10 kg was associated with a 12–13% increase in mortality. Similarly, two recent meta-analyses found that obesity was associated with a 41% increase in all-cause mortality and that overweight and obesity were associated consistently with treatment-related cardiotoxicity (42, 43). Although these studies refer to breast cancer survivors overall, we speculate that AA breast cancer survivors have a higher incidence of these chronic health conditions when compared to NHW breast cancer survivors. Thus, the disparities in survival between AA and NHW breast cancer survivors may be due partially to obesity and obesity-related consequences (e.g., comorbidities and cardiotoxicity).

##### Dietary intake

According to the American Cancer Society (ACS), cancer survivors should consume a diet high in fruits, vegetables, whole grains, and low in added sugars, red and processed meats (44). A meta-analysis of cohort studies indicated that adherence to dietary guidelines reduces cancer-specific mortality (45). Accordingly, cohort studies have found that participants adhering to the ACS dietary guidelines or a prudent diet experienced a 15–43% reduction in all-cause mortality and a 29% reduction in cancer recurrence (46–48). However, the protective effect of diet was not observed in all studies (46, 48).

Adherence to the American Cancer Society (44) dietary guidelines has not been studied extensively in AA breast cancer survivors. Paxton et al. (49) observed that AA breast cancer survivors consumed more fat, but consumed fewer daily servings of fruits and vegetables than NHW breast cancer survivors consume. Similarly, Ramirez et al. (50) found that 84% met guidelines for consumption of red and process meet, but most (80%) did not meet guidelines to fruit and vegetable consumption. Other studies in smaller samples of AA breast cancer survivors observed that more than two-thirds of AA breast cancer survivors exceeded the recommended daily intake of saturated fat, total fat, and added sugars (51, 52).

##### Physical activity

Physical activity has been associated with several benefits for cancer survivors across the cancer continuum (53–55) including a reduced risk of breast cancer-related mortality (56–59). In the Women's Healthy Eating and Living (WHEL) Study (59), women who were most active at baseline had a 53% lower mortality risk than those who were the least active. Similar results were observed for women in the Nurses' Health Study, (57) the Collaborative Women's Longevity Study (56), and the Health, Eating, Activity, and Lifestyle (HEAL) Study (60). Also, recent meta-analyses of controlled intervention trials of survivors showed that physical activity was associated with improvements in cardiorespiratory fitness, body mass index (BMI), percent body fat, upper and lower body strength, and health-related quality of life (54, 55). Similarly, positive improvements in health-related quality of life (HRQOL) were observed with resistance training (61, 62). Overall, physical activity is well tolerated during and after treatment and has minimal side effects (61, 63).

Despite the benefits associated with physical activity, many cancer survivors fail to meet current recommendations (≥30 min of moderate to vigorous physical activity on ≥5 days per week), and the rates of compliance have been lowest for AA breast cancer survivors (35, 49). In a secondary analysis of the WHEL study, Paxton et al. observed that 40% of AA breast cancer survivors met physical activity guidelines when compared to 60% of NHW breast cancer survivors (35). In the HEAL Study, only 24% of AA breast cancer survivors were compliant with guidelines (36, 64). Prior studies conducted in survivors recruited from community-based organizations have yielded higher compliance rates (65, 66). However, these rates may not be representative of AA breast cancer survivors nationwide.

Several studies have examined the relationship between physical activity and health-related quality of life in AA breast cancer survivors (35, 36). Physical activity has been associated consistently with physical function (35, 36, 65). In a sample of AA and Hispanic breast cancer survivors, Millet et al. (67) found that those who were physically active had better health-related quality of life scores than those who were not. Physical activity was also consistently associated with overall health-related quality of life and vitality (or energy/fatigue) (35, 36). Paxton et al. found that those meeting physical activity guidelines had better overall health-related quality of life, physical health, role limitations due to physical and emotional health, general health, mental health, and vitality than those who did not. Similarly, Smith et al. (65) found that those who met physical activity guidelines were better able to perform activities of daily living (e.g., going up and down stairs & performing household chores) and had lower levels of pain than those not meeting guidelines. Moreover, Diggins et al. (68) in a prospective study found those AA survivors who were physically active experienced greater improvements in mood as well as social, spiritual, and physical health than those who were not. Overall, the literature confirms associations between physical activity and both physical and mental health outcomes.

##### Sedentary behavior (SB)

SB can be defined as any “waking” activity characterized by energy expenditure between 0.75 and 1.5 metabolic equivalents and a sitting or reclining posture (69). SBs include, but are not limited to, screen time (i.e., television viewing or computer usage), riding in or driving an automobile, and sitting or reclining while engaged in leisure activities (i.e., reading, talking on the phone, eating, arts and crafts) (70). SB has emerged as an important risk factor for several chronic health conditions (e.g., breast cancer, heart disease, and diabetes) (71–75). In a sample of breast survivors, Phillips et al. (76) observed that with every quartile increase in sedentary time, mean levels of fatigue increased. Similarly, George et al. (77) using data from the HEAL Study observed that SB was associated with lower levels of physical functioning (β = −0.50, *p* = 0.03), general health (β = −0.75, *p* < 0.01), and overall physical health (β = −0.35, *p* < 0.01). We have not identified any studies that have examined the consequences of SB or breaks from prolonged sitting in AA breast cancer survivors. However, Paxton et al. (78) in a mixed methods study observed that AA survivors self-reported sitting up to 12.1 h per day for those who reported both leisure-time and work-related sitting. Additional research is needed to understand the antecedents and consequences of SB in both AA and NHW breast cancer survivors.

#### Intervention Studies in Breast Cancer Survivors

##### Weight loss studies in breast cancer survivors

Several studies have evaluated weight loss interventions in breast cancer survivors (79–84), and several more are ongoing (85–87). The Lifestyle Intervention study in Adjuvant Treatment of Early Breast Cancer (LISA) and the Exercise and Nutrition to Enhance Recovery and Good Health to You (ENERGY) trials were the largest (79, 80). The LISA trial compared the effectiveness of a telephone-delivered lifestyle intervention vs. a brief, mail-based intervention condition. The lifestyle condition lost approximately 5.5% of total weight compared to < 1% for the mail-based condition. Unfortunately, the LISA trial was terminated early due to loss of funding (88). The ENERGY trial was primarily a group-based study supplemented with telephone counseling calls and tailored newsletters. Weight loss at 12-months in the intervention condition was approximately 6% compared to 1.5% in the control condition, and significant differences were maintained at the 24-month follow-up assessment. Analyses are underway to determine the impact of the intervention on cancer recurrence and survival.

##### Weight loss intervention in AA breast cancer survivors

Few randomized weight loss interventions exist for AA breast cancer survivors (81, 89). In our review, we identified eight weight loss studies (52, 81, 89–94). Five of the eight studies used randomized designs (81, 89, 91–93). Four out of the eight resulted in statistically significant reductions in weight (52, 89, 92, 94). The Life Study (91) observed significant weight loss, but the change in weight was not significantly different from that of the comparison or control condition. Moving Forward II was the largest of the studies (89). Stolley et al. (89) found that a community-based weight loss intervention tailored to AA breast cancer survivors resulted in significant weight loss. The behavioral change strategies of Moving Forward II included self-monitoring, goal setting, self-efficacy, and social support. The remaining studies had sample sizes of < 45 participants. Of note, two of the studies measured cancer-related biomarkers (93, 94) and one used web-based monitoring with wireless/Bluetooth scales and activity trackers (93). Delgado-Cruzata et al. (94) did not observe changes in plasma biomarkers but was able to show that women who lost ≥2% of body fat experienced an improvement in the plasma biomarkers. Valle et al. (93) in a 3-arm trial using Facebook, wireless scales, and activity trackers, observed significant reductions in blood pressure. Overall, weight loss across the studies ranged from −1.9% (94) to −3.6% of weight (89). The weight loss studies were reported in Table 1.

**Table 1:** Identified weight loss intervention studies in African American breast cancer survivors.

**Study**	**Sample**	**Intervention**	**Outcomes**
Moving Forward II Stolley et al. (89)	Sample size: 246 AA Mean age: 58 years Mean BMI: 36 kg/m^2^ Mean years post-diagnosis: 7 years	Randomized to a 6-month weight loss intervention (interventionist-guided vs. self-guided) Social Ecological Model	Weight: −3.5 vs. −1.3%^*^ PA (minutes/week): +98.4 vs. +60.6 Diet (%Cal Fat): −2.1% vs. −0.7%
WELL Body Valle et al. (93)	Sample size: 35 AA Mean age: 53 years Mean BMI: 34 kg/m^2^ Mean years post-diagnosis: 3 years	Randomized to a 6-month self-regulation intervention with and without an activity monitor or a delayed intervention control Self-regulation Theory	Weight: −0.9 vs. −0.2 vs. +0.8% PA (Kcal/week): +432 vs. +72 Diet (%Cal Fat): not reported
Stepping STONE Sheppard et al. (81)	Sample size: 22 AA Mean age: 55 years Mean BMI: 36 kg/m^2^ Mean years post-diagnosis: 2 years	Randomized to a 12-week multimodal lifestyle intervention or a general health information program. Social Cognitive Theory (SCT) and Motivational Interviewing	Weight: −0.8 vs. +0.2% PA (minutes/week): +160 vs. +55 Diet (%Cal Fat): −4.8% vs. not reported
Mindful Eating Chung et al. (90)	Sample size: 22 AA Mean age: 50 years Mean BMI: 35 kg/m^2^ Mean years post-diagnosis: N/A	Single group into a 24-week diet and support group intervention using Cognitive behavior therapy (CBT) No stated health promotion theory.	Weight: −0.5%^*^ PA: N/A Diet (%Cal Fat): N/A
Curves II Delgado-Cruzata (94)	Sample size: 24 Hispanic, AA, and Afro-Caribbean survivors Mean age: 52 years Mean BMI: 33 kg/m^2^ Mean years post-diagnosis: 3 years	Randomized to a 6-month physical activity and dietary change intervention or a waitlist control group. No stated health promotion theory.	Weight: −2.0%^*^ PA: +85%,^*^ not in a standard format Diet (%Cal Fat): 4%
An Active Life Greenlee et al. (92)	Sample size: 42 Hispanic, AA, and Afro-Caribbean Mean age: 51 years Mean BMI: 33 kg/m^2^ Mean years post-diagnosis: 4 years	Randomized to a 6-month physical activity and dietary change intervention or a delayed intervention control. Stages of Change (SOC)	Weight: −3.3 vs. −1.8%^*^ PA: not in a usable format Diet (%Cal Fat): +0.5% vs. −0.9%
LIFE Study Djuric et al. (91)	Sample size: 31 AA Mean age: 56 years Mean BMI: 36.3 kg/m^2^ Mean years post-diagnosis: 6 years	Randomized to a 6-month dietician-led counseling, a spiritual counseling intervention or an unassigned control group. Social Cognitive Theory supplemented with the 12-Steps type curriculum	Weight: −2.5 vs. −1.5% PA (minutes/week): +144 vs. 0 Diet (%Cal Fat): −7.8% vs. −5.6%
Moving Forward I Stolley et al. (52)	Sample size: 23 AA Mean age: 51 years Mean BMI: 35 kg/m^2^ Mean years post-diagnosis: N/A	Single group into a 6-month weight loss intervention targeting dietary intake and physical activity Social Cognitive Theory and the Health Belief Model	Weight: −2.9%^*^ PA (minutes/week): +38.9 min/day Diet (%Cal Fat): −3.4%

* *denotes significant between-group differences for randomized designs or within-group changes over time in single group designs; AA, African American; ALIVE, A Lifestyle Intervention via email; % Cal fat, Percent of calories from fat; Body mass index (BMI), weight in kilograms (Kg)/height in meters squared (m^2^); Kcal, Kilocalorie; PA, Physical activity; N/A, not reported. Diet was reported in % calories of fat unless other results were described in the table. Weight loss was reported in % of weight loss. We focused on % calories from fat due to the findings from the Women's Intervention Nutrition Study. Outcomes were based on the pre- and post-test assessments and not follow-up assessments*.

##### Summary of identified weight loss intervention in AA breast cancer survivors

Overall, the weight loss studies conducted in AA breast cancer survivors have shown tremendous progress in the last decade. However, there is a need for more studies. In particular, future studies should: (a) consider examining cancer-related biomarkers, (b) ensure that studies are adequately powered to detect within- and between-group differences, and (c) use randomized designs. Research should also consider incorporating stepped-care or adaptive designs or those that provide more support for women who are not successful in the intervention condition. Future studies should also consider partner-based interventions to enhance the effectiveness of studies conducted in community-based settings (52). Daughters and Mothers Against Breast Cancer (DAMES) was a successful trial that recruited mother and daughter dyads (82). Interventions such as DAMES may help to combat the negative support that AA women experience in changing their diet and exercise behaviors.

##### Dietary intervention studies in breast cancer survivors

The Women's Intervention Nutrition Study (WINS) (95) and the Women's Health Eating and Living (WHEL) Study (96) were designed to test the impact of dietary interventions on breast cancer recurrence and survival. WINS emphasized a low-fat diet (≤ 15% of calories from fat), whereas the WHEL Study emphasized a diet high in fruits and vegetables (F&V; ≥8 servings/day) and fiber (≥30 g/day) and low in fat (≤ 20% of calories from fat). The WINS found that a 6 pound reduction in weight and a 19 gram reduction in dietary fat intake was associated with a ~24% lower risk for breast cancer recurrence. The reduction in breast cancer recurrence was more pronounced in women who had node-negative tumors, relative to women with other tumor characteristics (~42%) (95). Secondary analysis of the WHEL Study showed that a higher intake of F&Vs at baseline was associated with a reduced risk for breast cancer recurrence (*HR* = 0.7, 95% *CI* = 0.6–0.9), particularly for survivors who reported tamoxifen (HR = 0.6, 95% CI = 0.4−0.8) use (97). However, the WHEL Study dietary intervention did not yield significant improvements in cancer-related outcomes.

##### Diet trials in AA breast cancer survivors

We identified three dietary intervention studies that focused on AAs or included a sub-analysis of AAs (49, 98, 99). Secondary analysis of the WHEL study indicated that AA breast cancer survivors were able to increase their intake of fiber, fruits, and vegetables (49). Improvements in fiber and servings of fruits were maintained at the 4-year follow-up assessment. The WHEL study used a combination of intensive telephone counseling, newsletters, and cooking classing. Griffith et al. (98) used a culturally tailored version of WINS (i.e., WINS-C). The study resulted in a minor reduction of dietary fat consumption. WINS-C used a combination of coaching sessions with a registered dietician, group sessions, and follow-up telephone calls. Paxton et al. (99) in a randomized parallel-group design, observed positive trends in dietary intake, albeit not significantly different from that of the comparison group. Paxton et al. used a fully automated web-based program that utilized automated calls and encouraged goal setting and self-monitoring. Improvements in fruit and vegetable intake across the studies ranged from +25% (99) to +55% (49). Reductions in dietary fat intake across the studies ranged from 13% (49) to 31% (98).

##### Summary of dietary interventions in AA breast cancer survivors

Overall, dietary intervention studies in cancer survivors have not yielded promising results and have led many to believe that the relationships between diet and cancer prognosis may be inconclusive (100–102). Examining dietary patterns may help to uncover relationships that were not realized in interventions focused on intervening on a few nutrients or foods. Studies that personalize interventions to AAs and their food preferences are needed to enhance research in this population. Furthermore, there is a need to study the dietary patterns of AA breast cancer survivors and the role that specific patterns have on to cancer-specific outcomes. A summary of the dietary interventions was reported in Table 2.

**Table 2:** Identified diet only studies in African American breast cancer survivors.

**Study**	**Sample**	**Intervention**	**Outcomes**
ALIVE^∧^ Paxton et al. (99)	Sample size: 71 (59 AABCS) Mean age: 52 years Mean BMI: 31 kg/m^2^ Mean years post-diagnosis: 8 years	Randomized to a 3-month fully-automatic web- and email-based dietary or physical activity Social cognitive theory and goal setting	Weight: N/A PA (minutes/week): +49 vs. +94 min^*^ Diet (grams/day): −1.8 vs. −0.6 saturated fat grams
WINS-C Griffith et al. (98)	Sample size: 8 AA Mean age: 61 years Mean BMI: 31 kg/m^2^ Mean years post-diagnosis: 7 years	Diet only program–Culturally tailored Women's Intervention Nutrition Study	Weight: N/A PA: N/A Diet (% Cal fat): −3% Cal
WHEL Study Paxton et al. (49)	Sample size: 118 AA Mean age: 50 Mean BMI = 31 kg/m^2^ Mean years post-diagnosis: 2 years	Participants were randomized to low fat, high fiber diet vs. a standard national cancer institute diet. Social cognitive theory	Weight: −0.03 vs. + 0.75% PA: +47 vs. +45 Metabolic equivalent-minutes/week Diet (% Cal fat): −4% vs. −1.5%^*^

* *denotes significant between-group differences for randomized designs or within-group changes over time in single-group designs; AA, African American; ALIVE, A Lifestyle Intervention via email; % Cal fat, Percent of calories from fat; Body mass index (BMI), weight in kilograms (Kg)/height in meters squared (m^2^); PA, Physical activity; N/A, not reported; Diet was reported in % calories of fat unless other results were described in the table. Weight loss was reported in % of weight loss. We focused on % calories from fat due to the findings from the Women's Intervention Nutrition Study. Outcomes were based on the pre- and post-test assessments and not follow-up assessments. WHEL, Women's Healthy Eating and Living Study; WINS–C, Women's Intervention Nutrition Study–culturally tailored; ^∧^ALIVE was included in multiple tables because it was a parallel group design, which did not focus on weight loss as an outcome*.

##### Physical activity studies in breast cancer survivors

There are numerous studies in the literature outlining the benefits of regular physical activity for breast cancer survivors. Overall, these studies are positive and indicate that home- and community-based interventions are effective in improving physical activity (23, 103). More recently, there has been a push to examine the efficacy of physical activity on cancer-specific outcomes in various cancer populations (26). The data gathered from these studies will likely clarify the specific durations and frequencies of physical activity that produce desirable outcomes.

##### Physical activity studies in AA breast cancer survivors

Despite the push to expand research in the area of physical activity and cancer survivorship, only a few physical activity studies have focused on AA breast cancer survivors. We identified six studies that focused exclusively on improving physical activity (99, 104–108). One of the six studies incorporated diet but used a parallel group design (99), and one used yoga to improve quality of life (108). All studies resulted in significant improvements in physical activity. However, attendance was the outcomes of the yoga intervention. Wilson et al. (106) in an 8-week walking intervention observed significant weight loss. Strategies included using group- or team-based strategies (106, 107) or combined group and home-based strategies (104, 105). Spector et al. (104), in particular, used a certified personal training to encourage aerobic and resistance training. Paxton et al. (99) used a fully automated web-based program called A Lifestyle Intervention via Email (ALIVE). Improvements in physical activity ranged from +1.3% (106) to +544% (104). The physical activity studies we identified were reported in Table 3.

**Table 3:** Identified physical activity online intervention studies in African American breast cancer survivors.

**Study**	**Sample**	**Intervention**	**Outcomes**
Team walking Piacentine et al. (106)	Sample size: 12 Mean age: 54 years Mean BMI: 34 kg/m^2^ Mean years post-diagnosis: N/A	14-week team-based walking intervention. Theory of planned behavior	Weight: +1.1% PA (meters): +67 meters^*^ Diet: N/A
ALIVE∧ Paxton et al. (99)	Sample size: 71 Mean age: 52 years Mean BMI: 31 kg/m^2^ Mean years post-diagnosis: 8 years	Randomized to a 3-month fully-automatic web- and email-based dietary or physical activity Social cognitive theory and goal setting	Weight: N/A PA (minutes/week): +94 vs. +49^*^ Diet (grams/day): −0.6 vs. −1.8 saturated fat grams
Home-Based Spector et al. (104)	Sample size: 13 AA Mean age: 52 years Mean BMI: 30 kg/m^2^ Mean years post-diagnosis: N/A	Single group into a 16-week home-based physical activity intervention. No stated health promotion theory.	Weight:−0.3% PA (minutes/week): +212.7^*^ Diet: not reported
Gathering Place Nock et al. (105)	Sample size: 19 AA Mean age: 57 years Mean BMI: 32 kg/m^2^ Baseline weight: N/A Mean years post-diagnosis: N/A	Single group into a 20-week exercise and support group intervention.	Weight: +0.6% PA (meters/6-min): +135 meters^*^ Diet: not reported
Yoga Moadel et al. (108)	Sample size: 128, (54 AA) Mean age: 55 years Mean BMI: 34 kg/m^2^ Mean years post-diagnosis: 1.1	Randomized to a 12-week Yoga intervention or waitlist control condition. No stated health promotion theory.	Weight: N/A PA (yoga sessions): 7 ± 4 sessions Diet: N/A
Walking Program Wilson et al. (106)	Sample size: 22 AA Mean age: 55 years Mean BMI: 33 kg/m^2^ Baseline weight: 191 pounds Mean years post-diagnosis: N/A	Single group 8-week walking intervention Health Belief Model (HBM)	Weight: −1.1%^*^ PA (steps/week): +3,506 steps^*^ Diet: N/A

* *denotes significant between-group differences for randomized designs or within-group changes over time in single group designs; AA, African American; ALIVE, A Lifestyle Intervention via email; % Cal fat, Percent of calories from fat; Body mass index (BMI), weight in kilograms (Kg)/height in meters squared (m^2^); PA, Physical activity; N/A, not reported. Diet was reported in % calories of fat unless other results were described in the table. Weight loss was reported in % of weight loss. We focused on % calories from fat due to the findings from the Women's Intervention Nutrition Study. Outcomes were based on the pre- and post-test assessments and not follow-up assessments. ∧ALIVE was included in multiple tables because it was a parallel group design, which did not focus on weight loss as an outcome*.

##### Summary of physical activity studies in AA breast cancer survivors

Intervention studies have been largely successful in improving the physical activity habits of breast cancer survivors. Although few physical activity intervention studies exist for AA breast cancer survivors, they show positive results. Therefore, we can conclude that AA breast cancer survivors are capable of adhering to physical activity interventions that range from traditional home-based interventions to more complex web-based platforms. Future research should consider examining the benefits of strength training interventions in this population as well as the factors that mediate and moderate the relationships between physical activity and psychosocial, behavioral, and physiological outcomes.

##### Sedentary behaviors

To our knowledge, few intervention studies have intervened on sedentary time or have observed significant changes in sedentary time. In a web-based study of AA breast cancer survivors, Paxton et al. (99) observed significant reductions in sedentary time. The authors used a web-based platform entitled ALIVE. ALIVE is a fully automated system that uses weekly emails, self-monitoring, and goal-setting tools, and automated phone calls to improve health behaviors. The reduction in sedentary behavior was unexpected, as the ALIVE system targeted physical activity and mentioned SB as a health hazard. These results highlight the potential of a non-intensive or brief interventions to help breast cancer survivors adopt positive health behaviors. The ACTIVity And TEchnology (ACTIVATE) trial (109) will be one of the first to examine the effectiveness of a detailed curriculum to reduce prolonged sitting. The ACTIVATE trial will also incorporate activity trackers for self-monitoring and goal setting purposes. Additional studies that examine the impact of reducing time spent sitting on biological mediators, and cancer-related outcomes are warranted. Future studies should also consider the individual and joint effect of SB and physical activity interventions.

### Risk of Bias

A total of 7 interventions studies were characterized as high bias (52, 90, 98, 104–107), 7 studies were characterized a moderate bias (81, 91–94, 99, 108), and 2 studies were characterized a low bias (49, 89). Risk of bias was lowest for the weight loss studies and highest for the physical activity studies (see Table 4).

**Table 4:** Risk of bias ranking for the identified intervention studies.

**Study author, year**	**Sample**	**Design**	**Outcome**	**Total score**	**Bias ranking**
**WEIGHT LOSS**					
Stolley et al. (89)	>50	Randomized	Objective	99	Low bias
Valle et al. (93)	< 50	Randomized	Objective	88	Moderate bias
Sheppard et al. (81)	< 30	Randomized	Objective	77	Moderate bias
Chung et al. (90)	< 30	Single group	Self-reported	33	High bias
Delgado-Cruzata et al. (94)	< 30	Randomized	Objective	77	Moderate bias
Greenlee et al. (92)	< 50	Randomized	Objective	88	Moderate bias
Djuric et al. (91)	< 30	Randomized	Objective	88	Moderate bias
Stolley et al. (52)	< 30	Single group	Objective	55	High bias
**DIETARY INTAKE**					
Paxton et al. (99)	>50	Randomized	Self-reported	77	Moderate bias
Griffith et al. (98)	< 30	Single group	Objective	55	High bias
Paxton et al. (49)	>50	Randomized	Objective	99	Low bias
**PHYSICAL ACTIVITY**					
Piacentine et al. (107)	< 30	Single group	Objective	55	High bias
Paxton et al. (99)	>50	Randomized	Self-reported	77	Moderate bias
Spector et al. (104)	< 30	Single group	Objective	55	High bias
Nock et al. (105)	< 30	Single group	Objective	55	High bias
Moadel et al. (108)	>50	Randomized	Self-reported	77	Moderate bias
Wilson et al. (106)	< 30	Single group	Objective	55	High bias

*Sample sizes of <30, 30 to 49, and 50 or higher were characterized as low, moderate, and high quality, respectively. Randomized trials were characterized as high quality, while other designs were characterized as low quality. Objectively assessed anthropometric, dietary, and physical activity outcomes were characterized as high quality, whereas self-reported outcomes were characterized as low quality. Each rating was transformed to a numerical, whereby scores of 11, 22, and 33 characterized as low, moderate, and high quality. Scores were summed across categories. Total scores of < 60, 60 to < 99, and 99 were characterized as high, moderate, and low bias studies*.

## Discussion

### Summary of Main Findings

Our review indicated that many AA breast cancer survivors failed to meet guidelines for healthy living as indicated by high rates of obesity, poor diet, and physical inactivity. Although many of the studies were rated as having a high or moderate risk of bias, many resulted in significant improvements in health behaviors. The majority of the studies had small samples sizes, which resulted in significant bias. Only two of the studies were rated as having low bias. Notably, Stolley et al. (89) demonstrated that guided community-based trials could be successful in producing significant weight loss. Additional robust randomized trials are needed.

#### Challenges to and Solutions for Improving Minority-Based Survivorship Research

Studies have indicated that AA breast cancer survivors experience significant social (e.g., support), environmental (e.g., access, crime, neighborhood cohesion), and personal barriers (e.g., time and interest) that may diminish their ability to engage in recommended levels of activity or consume a healthy diet (110–112). Other challenges such as poor patient-provider communication and that the failure of physicians to provide equitable care often contributes to mistrust among AA breast cancer survivors (113, 114). Comprehensive strategies that reach AA breast cancer survivors where they are and provide evidence-based strategies may help to reduce their actual and perceived barriers. AA women may also benefit from removing themselves from environments and people that are not supportive of their desired lifestyle changes.

Demark-Wahnefried et al. (26) recommended collaborating with community-based organizations, utilizing local farmer's markets, and promoting the 5-A's (Ask, Advise, Assess, Assist, and Arrange) in a clinical setting as potential strategies to reduce health-related barriers. In AA communities, efforts can be made to leverage churches, civic organizations, parks, and recreation centers to provide support systems and venues for programmatic activities. Expanding upon models such as Body and Soul (115, 116) may also hold promise in promoting physical activity and weight-management in community-based settings. Similar efforts are underway in underserved populations in Hawaii (117). These efforts are promising because they incorporate traditional foods and spirituality to encourage behavior change. In addition, reducing prolonged sitting or breaking up sedentary time may be a low hanging fruit (78, 118, 119). Capitalizing on concepts such as “tiny habits” proposed by BJ Fogg at Stanford University may also provide sufficient motivation to make long-lasting behavioral changes (120). Furthermore, focusing on enjoyable activities such as gardening (121), dancing (122), and acquiring extra steps while shopping may be additional strategies to promote.

#### The Need for Comprehensive and Personalized Approaches

Literature documenting the dietary habits of AA breast cancer survivors has been limited to single nutrient analyses (e.g., fruit and vegetable consumption and dietary fat intake). Single nutrient analyses may be sufficient for understanding what nutrients are needed, but not sufficient for describing overall dietary patterns or diet quality. Investigators have been studying dietary patterns and diet quality indexes as a means to summarize entire diets rather than single nutrients (123–125). Researchers interested in understanding the role that diet plays in the overall health and well-being of AA breast cancer survivors should consider applying similar techniques. Studies conducted in primarily NHW samples have indicated that a prudent diet (i.e., colorful fruits & vegetables, whole grains, nuts, and cereals) rather than a Western diet (i.e., red and processed meats, foods high in fat and sugar, and refined grains) was associated with overall survival in cancer survivors (47). Large-scale and randomized controlled studies are needed to determine whether dietary patterns or indices influence the health of AA breast cancer survivors (24).

Similarly, additional studies are warranted to determine whether AA breast cancer survivors may benefit more from different physical activity frequencies, intensities, types, and durations. There may also be a need for physical activity interventions that are more personalized (126, 127). Jones et al. (127) indicated that exercise prescription often adopts a one-size-fits-all approach, which may not be ideal for AA breast cancer survivors whom frequently indicate significant perceived and actual barriers to physical activity (110, 111). AA breast cancer survivors are often not interested in or motivated enough to engage in regular physical activity. Interventions that take into account the individual preferences of AA women are needed.

#### The Potential for Internet and Other Web-Based Platforms

We identified two studies that used web-based approaches. Both had significant strengths and weaknesses. ALIVE was plagued with functionality challenges that lowered adherence rates (99), whereas the WELL Body study was not powered adequately to detect differences between conditions (93). Despite the limitations of these studies, technology-enhanced approaches offer some advantages, including the opportunity for individual tailoring, elimination of barriers (i.e., cost, time, people, distance); and sustained motivation and feedback for extended periods (128). We would be remiss if we did not indicate the limitations of web-based approaches. The digital divide (i.e., computer literacy and internet access) still exist by race and socioeconomic position, and access to the internet is still less stable for racial/ethnic minorities as well as rural populations (129). Additionally, those who are financially stable may experience periods of economic insecurity attributable to their health status (130, 131); these occurrences may not allow them to participate fully and interventions that rely on web-based technologies like Wi-Fi connections. Thus, researchers that rely on smartphones and their related apps may be able to overcome some of these technological disparities, but may encounter other phone-related challenges (i.e., data, screen size, vision, etc.).

#### Opportunities for Analytical Advancements

There are new frontiers that should be explored to advance the field of cancer survivorship (132). Questions remain in the areas of identifying (a) effect modifiers, (b) subpopulations that respond differently, and (c) identifying the optimal physical activity prescription and dietary patterns. The questions proposed by Courneya et al. (132) can be expanded to include specific dietary components and interactions that exist among multiple lifestyle behaviors, sociodemographic, and medical characteristics. Techniques such as Random Forest (133) are capable of analyzing high dimensional data with small sample sizes. Others have applied similar techniques to study PA patterns (134). Other intuitive methods such as clustering and recursive partitioning are capable of identifying subgroups with similar behavioral patterns (135, 136). Paxton et al. (137) utilized recursive partitioning to examine correlates of physical activity in AA breast cancer survivors. This approach can be expanded to include time to event outcomes.

## Limitations

In this review, we conducted a comprehensive search to identify peer-reviewed articles. However, we did not select unpublished dissertations and theses or published peer-reviewed abstracts related to our search terms. In addition, we excluded articles they were not seminal in the field of cancer survivorship as well as many articles that focused primarily on psychosocial outcomes. Furthermore, many of the intervention studies we identified were rated as having high or moderate levels of bias. Larger studies are needed to confirm the strategies applied in several studies. Despite our limitations, we found no similar reviews to compare our findings. Thus, this study represents a unique contribution to the literature.

## Conclusion

Our review provides objective information identifying the strengths, weaknesses, and opportunities that exist for designing lifestyle interventions and epidemiological studies for AA breast cancer survivors. AA breast cancer survivors, like other vulnerable cancer populations, are often difficult to track and trace. Therefore, innovative strategies are needed to recruit and retain them in randomized trials. Leveraging partnerships with community-based organizations, support groups, and health systems may help to identify sufficient numbers to support a clinical trial. Studies are warranted that examine the impact of lifestyle behaviors in individuals with multiple comorbid conditions, as many minority survivors are predisposed to higher rates of diabetes and cardiovascular disease when compared to other ethnic groups. Lastly, reducing sedentary behavior represents a “tiny habit” that may result in sustainable changes in physical activity over time.

## Author Contributions

RP conceived the study. WG and GL reviewed and summarized intervention studies and created tables. RP, WG, GL, LD, and KA-W drafted and edited versions of the manuscript.

### Conflict of Interest Statement

The authors declare that the research was conducted in the absence of any commercial or financial relationships that could be construed as a potential conflict of interest.
